# Rickettsial Cerebellitis: A Rare Neurological Manifestation

**DOI:** 10.7759/cureus.42901

**Published:** 2023-08-03

**Authors:** Sai Sri Venkata Yeshwanth Damalapati, Raghavendra Deshpande, Narayana S Moola, Rakesh H Jadav, Sunil Havannavar

**Affiliations:** 1 Neurology, Manipal Hospital, Bengaluru, IND; 2 Critical Care Medicine, Manipal Hospital, Bengaluru, IND; 3 Internal Medicine, Manipal Hospital, Bengaluru, IND

**Keywords:** orientia tsutsugamushi, infectious disease, tropical illness, scrub typhus, cerebellitis

## Abstract

Scrub typhus is a vector-borne disease caused by gram-negative bacilli, *Orientia tsutsugamushi*. The vector of scrub typhus is the mite. The clinical manifestations often present with either a simple fever or life-threatening multi-organ dysfunction. Neurological manifestations also vary, and the incidence of neurological manifestations is unknown. Cerebellitis is one of the rare neurological manifestations associated with scrub typhus. In this case report, we present the case of a 35-year-old man who tested positive for scrub typhus (IgM enzyme-linked immunosorbent assay (ELISA) blood test) with a history of fever and cerebellar signs and symptoms. He was managed with antibacterial agents and made a good recovery.

## Introduction

Scrub typhus is a tropical fever disease that has traditionally been more prevalent in rural areas but is increasingly affecting urban populations, particularly in Southeast Asian countries such as India [1,2]. The clinical presentation of this rickettsial disease can vary widely, ranging from mild fever to potentially life-threatening complications such as acute liver failure, renal failure, acute respiratory distress syndrome, myocarditis, septic cardiomyopathies, secondary hemophagocytic lymphohistiocytosis (HLH), and disseminated intravascular coagulation (DIC) [3-7]. Unusual clinical manifestations of scrub typhus are also becoming more common. The diagnosis of scrub typhus is arrived at by detecting IgM antibodies through an enzyme-linked immunosorbent assay (ELISA), which is now widely accepted due to its high sensitivity and specificity. The drug of choice for scrub typhus treatment is doxycycline. However, a recent study in India showed that combining azithromycin and doxycycline may be more effective in treating severe infections [8].

## Case presentation

A 35-year-old male, an English teacher by occupation at a high school in a village near Bengaluru, India, presented with incoordination and slurred speech for three days. A week before his current admission, he had a five-day hospitalization for fever, vomiting and diarrhea, burning micturition, and increased urinary frequency. There was no blood in stools or hematemesis. His aspartate transaminase (AST) and alanine transaminase (ALT) levels were mildly elevated. He had a medical history of varicella-zoster encephalitis 10 years ago, from which he recovered completely. He had been taking phenytoin 100mg twice daily, which was later changed to sodium valproate 500mg twice daily for two years, which was then gradually tapered and eventually stopped. He had no family history of neurological conditions.

**Figure 1:**
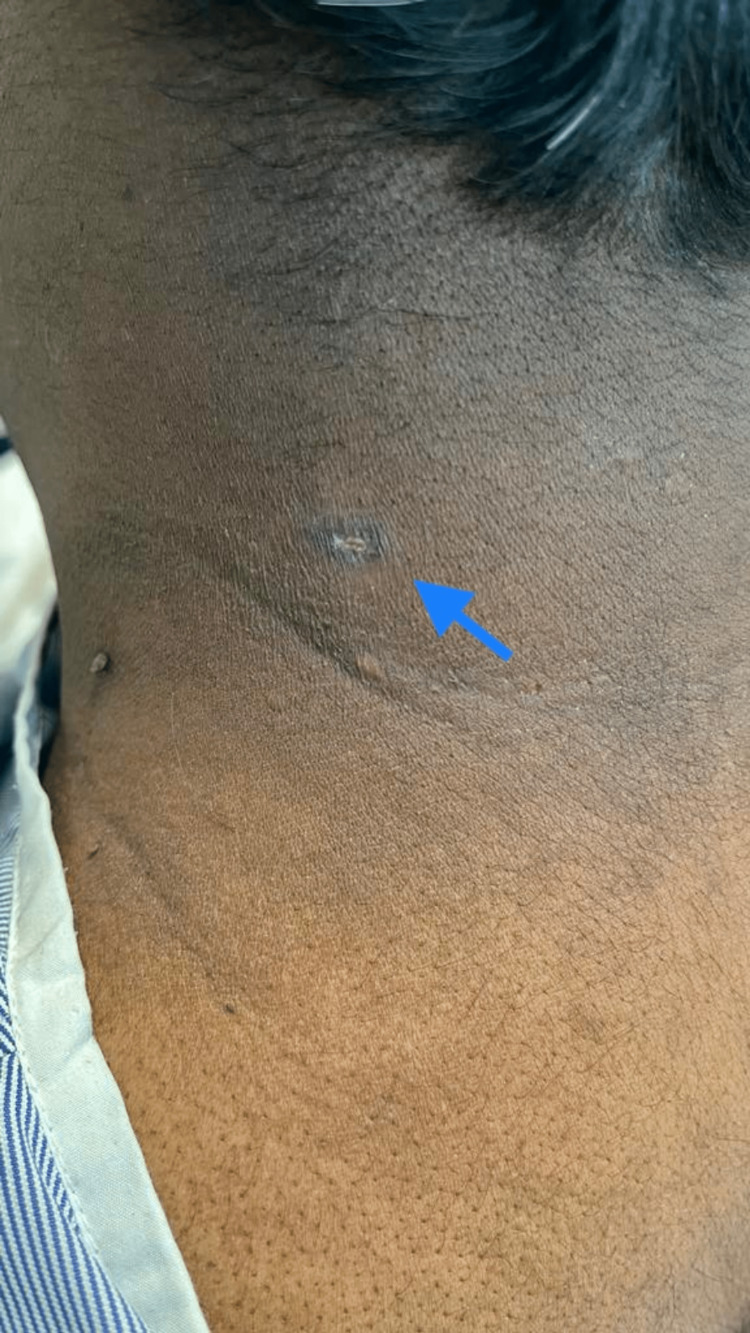
Site of the infected chigger bite

On admission to our hospital, the patient's blood pressure (BP) was 90/50mmHg with a heart rate of 115 and a temperature of 100.4 Fahrenheit requiring resuscitation with IV fluids. A healing maculopapular rash was noted on the left posterior surface of his neck (Figure 1). This rash started around seven to eight days before the current admission. This rash and his lab reports of transaminitis and thrombocytopenia raised suspicion of a tropical infection.

Neurological examination revealed cerebellar-type dysarthria, pan-directional nystagmus, and truncal ataxia. Limb ataxia was also present, manifested as dysmetria, and intention tremors (large amplitude, low frequency) in his finger-nose and heel-shin testing. Other cerebellar signs were present, such as dysdiadochokinesia (slow and clumsy alternating movements) (Video 1).

**Video 1:** Cerebellar signs in our patient (nystagmus, impaired finger-nose testing, dysdiadochokinesia, gait ataxia)

On investigation, he had a serum WBC count of 11,100/μL with lymphocyte predominance and a drop in platelet count from 404,000 to 168,000/cu.mm. His AST and ALT were 90 IU/L and 213 IU/L, respectively. His CSF showed lymphocytic pleocytosis (8 cells/cu.mm) with a few RBCs in the background and a protein of 64.5 mg/dl. Serum samples were sent for a tropical infection workup which showed a positive ELISA IgM for scrub typhus; other biochemical/autoimmune investigations were unremarkable. His MRI brain did not reveal any abnormalities. He was treated with a pulse dose of methylprednisolone (1g IV) for three days, doxycycline (100mg twice daily), and other supportive measures in the ICU. On the third day, he was afebrile, and his cerebellar signs and platelet count improved. Doxycycline was stopped after 14 days; he was discharged in the second week of admission. There was mild improvement in ataxia on the day of discharge, with a Barthel index of 55 (partially dependent), an improvement from a score of zero at the time of presentation to our hospital. On the 30th day follow-up, his ataxia significantly improved, and he scored a Barthel index of 90 (Video 2).

**Video 2:** Improvement of cerebellar signs in our patient at the one-month follow-up

## Discussion

Scrub typhus is a severe zoonotic ailment affecting nearly one billion people in endemic areas, resulting in one million new cases diagnosed yearly [9]. Patients may present with a range of clinical manifestations, and complications typically arise after the first week of illness, including acute renal failure, acute respiratory distress syndrome (ARDS), septic shock, jaundice, myocarditis, meningoencephalitis, etc. Neurological manifestations of scrub typhus are common and can include meningitis, encephalitis, cranial nerve involvement, stroke, cerebral vein thrombosis, cerebellitis, isolated 6th and 7th cranial palsy, delirium, trigeminal neuralgia, opsoclonus, myoclonus, polyneuropathy, Guillian Barre syndrome, Parkinsonism (transient), acute disseminated encephalomyelitis (ADEM), and brachial plexopathy [10,11]. Pathological examination of the central nervous system may show clusters of microglial cells and focal mononuclear cell exudates in the leptomeninges. Although neurological involvement is relatively common, focal neurological deficits are rare [12]. The CSF samples from patients with scrub typhus show mild-to-moderate lymphocytic pleocytosis, normal glucose levels, and a slight increase in CSF protein. The presence of RBCs in the CSF samples also suggests the presence of generalized vasculitis. Pai et al. showed the presence of the rickettsial pathogen in the CSF samples of patients suffering from scrub typhus with the help of nested polymerase chain reaction (PCR) [12]. 

Cerebellitis is a rare but significant complication of scrub typhus that presents as acute cerebellar dysfunction, including ataxia, dysarthria, and nystagmus. The time interval between prodromal symptoms and cerebellar signs suggests an immune-mediated response with resultant vasculitis rather than a direct infection. The diagnosis is made using serological testing for scrub typhus, but CSF PCR can also be used. Doxycycline is the mainline therapy for rickettsial infections. However, a recent study in India showed that combining azithromycin and doxycycline may be more effective in treating severe infections [8].

It is essential to rule out other treatable causes of acute to subacute cerebellar involvement, such as neurosarcoidosis, paraneoplastic encephalitis, etc., all of which may respond to steroid therapy and/or intravenous immunoglobulins. There are currently no guidelines for the use of steroids in rickettsial disease. Choi et al. administered steroids to a patient with rickettsial disease who presented with progressive neurological symptoms, including restlessness, ophthalmoplegia, and upper limb paralysis. The authors observed a notable improvement in the patient's neurological symptoms, and no new neurological complications were reported [13]. In our case, the decision to administer steroids was made after weighing the benefits and risks and discussing them with the patient and family.

Our patient showed significant neurological improvement following steroid therapy. However, given the limited number of reported cases of steroid therapy in rickettsial disease, particularly in cases with neurological involvement, further studies are necessary to determine the optimal approach to treatment.

## Conclusions

Cerebellitis resulting from scrub typhus is a rare but significant complication of this zoonotic ailment. Prompt identification, along with the administration of appropriate antibiotics, steroids, and neurological support, can lead to complete recovery. Therefore, it is essential for healthcare providers to be aware of the potential neurological complications associated with scrub typhus, especially in regions where it is endemic. 

## References

[1] Silpapojakul K (1997). Scrub typhus in the Western Pacific region. Ann Acad Med Singapore.

[2] Chrispal A, Boorugu H, Gopinath KG (2010). Acute undifferentiated febrile illness in adult hospitalized patients: the disease spectrum and diagnostic predictors - an experience from a tertiary care hospital in South India. Trop Doct.

[3] Chung JH, Lim SC, Yun NR, Shin SH, Kim CM, Kim DM (2012). Scrub typhus hepatitis confirmed by immunohistochemical staining. World J Gastroenterol.

[4] Vikrant S, Dheer SK, Parashar A (2013). Scrub typhus associated acute kidney injury—a study from a tertiary care hospital from western Himalayan State of India. Ren Fail.

[5] Saxena A, Khiangte B, Tiewsoh I (2014). Scrub typhus complicated by acute respiratory distress syndrome and multiorgan failure; an unrecognized alarming entity in central India: a report of two cases. J Family Med Prim Care.

[6] Sittiwangkul R, Pongprot Y, Silviliarat S, Oberdorfer P, Jittamala P, Sirisanthana V (2008). Acute fulminant myocarditis in scrub typhus. Ann Trop Paediatr.

[7] Ono Y, Ikegami Y, Tasaki K, Abe M, Tase C (2012). Case of scrub typhus complicated by severe disseminated intravascular coagulation and death. Emerg Med Australas.

[8] Varghese GM, Dayanand D, Gunasekaran K (2023). Intravenous doxycycline, azithromycin, or both for severe scrub typhus. N Engl J Med.

[9] Xu G, Walker DH, Jupiter D, Melby PC, Arcari CM (2017). A review of the global epidemiology of scrub typhus. PLoS Negl Trop Dis.

[10] Gulati S, Maheshwari A (2013). Neurological manifestations of scrub typhus. Ann Indian Acad Neurol.

[11] Mahajan SK, Bakshi D (2007). Acute reversible hearing loss in scrub typhus. J Assoc Physicians India.

[12] Pai H, Sohn S, Seong Y, Kee S, Chang WH, Choe KW (1997). Central nervous system involvement in patients with scrub typhus. Clin Infect Dis.

[13] Choi HC, Wie SH, Lee SY (2002). Use of high-dose steroid in a case of scrub typhus with acutely progressive local neurologic symptoms. Korean J Infect Dis.

